# X-Ray Solution Scattering of Squid Heavy Meromyosin: Strengthening the Evidence for an Ancient Compact off State

**DOI:** 10.1371/journal.pone.0081994

**Published:** 2013-12-17

**Authors:** Richard E. Gillilan, V. S. Senthil Kumar, Elizabeth O'Neall-Hennessey, Carolyn Cohen, Jerry H. Brown

**Affiliations:** 1 Macromolecular Diffraction Facility, Cornell High Energy Synchrotron Source, Ithaca, New York, United States of America; 2 Rosenstiel Basic Medical Sciences Research Center, Brandeis University, Waltham, Massachusetts, United States of America; University of Oulu, Finland

## Abstract

The overall conformations of regulated myosins or heavy meromyosins from chicken/turkey, scallop, tarantula, limulus, and scorpion sources have been studied by a number of techniques, including electron microscopy, sedimentation, and pulsed electron paramagnetic resonance. These studies have indicated that the binding of regulatory ions changes the conformation of the molecule from a compact shape found in the “off” state of the muscle to extended relationships between the tail and independently mobile heads that predominate in the “on” state. Here we strengthen the argument for the generality of this conformational change by using small angle X-ray scattering on heavy meromyosin from squid. Small angle X-ray scattering allows the protein to be visualized in solution under mild and relatively physiological conditions, and squid differs from the other species studied by at least 500 million years of evolution. Analysis of the data indicates that upon addition of Ca^2+^ the radius of gyration increases. Differences in the squid “on” and “off” states are clearly distinguishable as bimodal and unimodal pair distance distribution functions respectively. These observations are consistent with a Ca^2+^-free squid heavy meromyosin that is compact, but which becomes extended when Ca^2+^ is bound. Further, the scattering profile derived from the current model of tarantula heavy meromyosin in the “off” state is in excellent agreement with the measured “off” state scattering profile for squid heavy meromyosin. The previous and current studies together provide significant evidence that regulated myosin's compact off-state conformation is an ancient trait, inherited from a common ancestor during divergent evolution.

## Introduction

**Figure 1:**
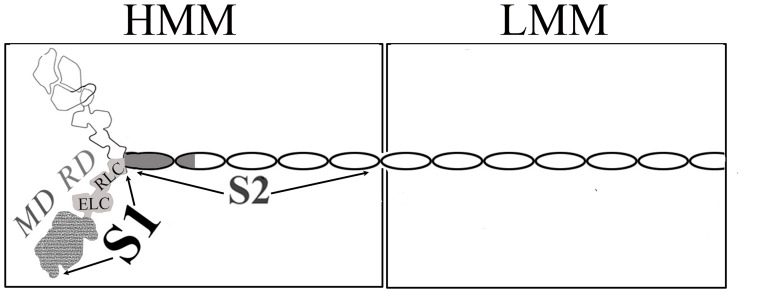
Schematic figure of a myosin dimer showing location of domains. The structures of regions with variable shades of grey have been determined to atomic resolution.

Myosin is most often in an “off” state. Vertebrate striated muscle contraction is regulated by the thin filament's troponin/tropomyosin complex. By contrast, myosin itself (see Fig. 1) controls contraction in invertebrate striated muscle, molluscan muscles, and in many vertebrate smooth muscles, through changes in its conformation. (For review see [1]; see also [2], [3]). These regulated myosins have different activation triggers: the direct binding of Ca^2+^ to the essential light chain (ELC) (stabilized by the regulatory light chain, RLC) in mollusks [4], and the phosphorylation of the RLC in vertebrate smooth muscles and invertebrate striated muscles (for review, see [5]). Whereas the on state can be achieved with a single myosin head (subfragment 1, or S1), a far larger portion of the regulated myosin molecule is required for a functional off state. The two-headed heavy meromyosin (HMM) is the minimal unit capable of regulation [6], [7], [8], and is soluble in low salt solutions due to the removal of the filament-forming portion of its alpha-helical coiled-coil tail (See Fig. 1 for schematic of myosin structure).

Despite these different triggers for contractile activity in regulated myosins, both regulatory ions appear to produce a similar conformational change: from a generally compact off-state conformation, where the two heads are folded back towards the tail and contact each other asymmetrically, to an on state where the tail and independently mobile heads have a somewhat more extended relationship. Compact shapes of the off state of HMM, myosin, and/or the thick filament have been viewed directly, and in some cases to ∼20 Å resolution, by electron microscopy [2], [9], [10], [11], [12], [13], [14], [15]. The difference in the overall shape of the on and off states was determined by sedimentation studies of scallop myosin, where, in the presence of ADP or ATP analogs, Ca^2+^ yields a slower sedimentation velocity than that observed in the presence of EGTA [16]. A similar change in vertebrate smooth muscle myosin has also been shown recently by pulsed EPR studies; here, in the unphosphorylated state, compact off state conformations coexist with conformations where the heads are splayed out, and this equilibrium is shifted away from the compact conformation upon phosphorylation [17].

No atomic resolution structure of the myosin dimer or of HMM is available, and questions also remain concerning the current low-resolution descriptions. A technical concern, for example, is the conditions under which the compact conformation has been determined. The sedimentation, electron microscopy or EPR techniques that have been employed subject the protein to relatively unphysiological and/or extreme conditions. Sedimentation uses high g forces, and negative staining or freezing have been employed for the electron microscopy and EPR experiments. Another question about the compact off-state conformation involves its exact role in evolutionary biology. Although this conformation has been shown to be present in species separated by 600 million years of evolution [12], it has yet to be determined whether this conformation is more likely an ancient trait [12], hence descended from a common ancestor during divergent evolution, or whether it more likely arose by convergent evolution. Compact conformations have been reported for myosins (or HMM) from five diverse species: chicken/turkey [9], [10], [11], [12], scallop [12], tarantula [2], [13], limulus [2], [14], and scorpion (see ref. [15]), and such a collection represents a moderately sized sample for inferring the prevalence of this conformation throughout evolutionary history.

We have now extended these studies to another species and have used another biophysical technique, with the aim of assessing both the current picture of how myosin changes its shape during regulation, as well as the evolutionary history of myosin-linked regulation. These studies involve examining the active and off-state conformations of HMM in solutions of physiological buffers using small angle X-ray scattering (SAXS). Moreover, these experiments were performed on HMM from squid, which is separated by at least 500 million years of evolution from any of the other species for which studies of the off-state myosin have been described previously. Our results and analyses have fortified our understanding of the overall conformational change that occurs for myosin upon activation.

## Results

Radius of gyration (R_g_) and intensity at zero scattering angle (I(0)) were computed using the Guinier method as implemented in RAW (Table S1)[18]. In the range qR_g_<1.3, squid HMM preparations show evidence of some aggregation at the smallest angles but present significant concentration-independent linear regions from which R_g_ values can be extracted (Figs. S1, S2). In the “off” state (i.e., with EGTA, see Methods), the Rg as determined by the Guinier method is 59.6±0.8 Å. The inverse Fourier transform (IFT) method described below yields an independent estimate of R_g_ = 59.7±0.2 Å at maximum diameter 200 Å (see Methods). Using the same analysis, the “on” state yields a Guinier-derived R_g_ value of 77±2 Å. The IFT analysis gives a somewhat higher value of 83.9±0.8 Å with maximum diameter of 290 Å. The “on” state is systematically larger than the “off” state as measured by R_g_ regardless of maximum diameter setting chosen.

For both on and off states, no significant concentration effects can be observed within the expected error bounds in these dilute solutions (σ/Rg for first exposures are 1.4% for the “off” state, and 2.6% for the “on” state), nor was any radiation damage observed upon comparing curves of different exposures (Table S1). Kratky plots of both states show fall-off to zero intensity at wide angle (the Porod region), which is characteristic of folded proteins (Fig. S3).

Molecular weight estimates based on a glucose isomerase standard (1.25 mg/ml) give 268±45 kDa for the “off” state and 241±12 kDa for the “on” state. Independent molecular weight estimates based on a corrected Porod volume give 317 kDa for the “off” state and 262 kDa for the “on” state [19]. The “off” state values are in reasonable agreement with the value of 282 kDa calculated for tarantula HMM as used in the profile comparison described below. The “on” state estimates appear to be systematically low.

**Figure 2:**
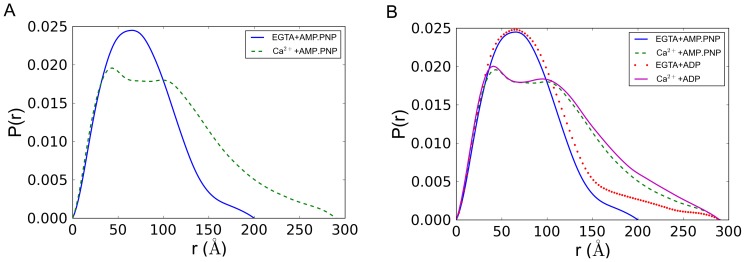
Pair distance distribution function of squid HMM derived from small angle x-ray solution scattering data. The pair distance distribution function (denoted P(r)) function may be interpreted as the r^2^-weighted distribution of all possible electron pair distances within the protein. **A**) In the absence of calcium (EGTA+AMP.PNP), the protein assumes a unimodal P(r) distribution characteristic of a simple compact globular shape (solid line). In the presence of calcium (Ca^2+^+AMP.PNP) the protein gives a more extended bimodal P(r) distribution indicating the presence of two separated domains (dashed line). **B**) Similar P(r) functions are observed when ADP is used instead of AMP.PNP. For comparison, curves are shown together with the same maximum diameter cutoff (Dmax) of 300 Å.

The pair distance distribution functions, denoted P(r), for both states were calculated using the inverse Fourier method as implemented in the GNOM program [20] (Fig. 2). The P(r) function is the SAXS analog of the Patterson function popular in conventional crystallography and can be understood as an r^2^-weighted distribution of all possible electron pair distances within the protein. The maximum diameter object observable in this experiment is set by the Shannon Limit π/qmin = 312 Å where qmin is the minimum observable q value. In the absence of calcium (EGTA+AMP.PNP), the protein assumes a unimodal P(r) distribution characteristic of a simple compact globular shape (solid line in Fig. 2a). In the presence of calcium (Ca^2+^+AMP.PNP) the protein gives a more extended bimodal P(r) distribution indicating the presence of two separated domains (dashed line in Fig. 2a). As a check of the IFT solution quality, the back transforms of the IFT solutions are shown to be in good agreement with the experimental data (Figs. S4 and S5).

**Figure 3:**
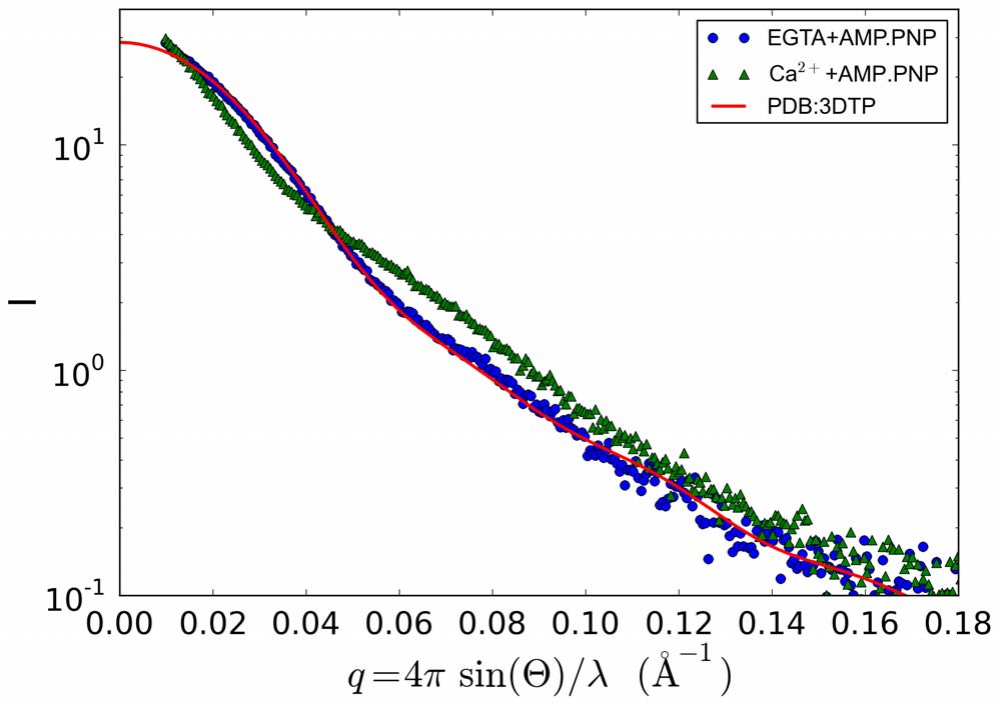
Comparison of model-based and measured SAXS profiles. The predicted scattering profile is based on an electron microscopy derived off-state tarantula HMM model. Integrated scattering intensity (I in arbitrary units) is given as a function of momentum transfer (q in Å^−1^, approximately proportional to the scattering angle). The model used is PDB i.d. 3DTP, with the deletion of the 50 N-terminal residues of the RLC unique to this tarantula myosin. The fit of the PDB computed profile to the squid HMM EGTA profile is quite good (slight aggregation is noted at smallest q), and is much better than the fit to the squid HMM Ca2+ data.

A low resolution atomic model of tarantula HMM in the “off” state has been published based on cryo-electron microscopy 3D image reconstruction (PDB ID 3DTP) [13]. To facilitate comparison, we removed the first 50 residues of each original tarantula regulatory light chain to give a sequence with molecular weight 282 kDa. Comparison of the calculated scattering profile of tarantula HMM with the measured profile for Ca^2+^-free (EGTA) squid HMM shows excellent agreement (Fig. 3). The Ca^2+^ squid HMM profile is also shown for comparison.

Shape reconstructions show that the Ca^2+^ squid HMM is elongated and somewhat narrower, but otherwise similar to the non-Ca^2+^ case (Fig. S6). Comparison of scattering curves generated from bead models with the experimental data are given in Figs. S7 and S8. Further details of the IFT and shape reconstructions are given in Methods S1.

## Discussion

Squid is a rising star in the field of myosin structure. Although it is a mollusk, as is scallop (which had been the species of choice in this field), squid is quite distinctive. Squid and scallop belong to different molluscan classes: scallop is a bivalve, whereas squid is a cephalopod — a uniquely “intelligent” class of mollusc. These two classes diverged from one another about 500 million years ago [21]. The sequences of their myosins, although homologous, show numerous differences that reflect this evolutionary distance. For example, upon comparing GI:3252880 and GI:5612 using BLAST, we see that 26% of these squid and scallop heavy chain sequences are different from one another. Such differences between squid and scallop have in turn affected the nature of their respective studies. Despite much effort with scallop in this laboratory, only squid has yielded a highly ordered crystal structure of the head domain of a myosin II in a fully closed cleft rigor–like state [22]. Similarly, while efforts at crystallizing scallop heavy meromyosin (HMM) in the off state have been unsuccessful, we have been able to obtain crystals of squid HMM that currently diffract to about 5 Å resolution in certain directions [23], and are attempting to improve the resolution as well as to phase the X-ray diffraction data.

Here we have used SAXS to determine whether squid HMM changes its overall shape in response to its regulatory ion as observed in scallop and in other non-molluscan species using diverse techniques. A compact off-state structure has been indicated by sedimentation experiments [16], as well as by electron microscopic investigations of negatively stained molecules [2], [11], [12], frozen crystals [9], [10], and filaments[13], [14]. In contrast to these methods, SAXS allows the protein to be visualized under mild and relatively physiological conditions. Here the protein is in solution, and is not subjected to any unphysiological forces (such as crystal packing or high g-forces), temperatures, or extraneous chemical agents (fixatives or crystallization agents) required for the other techniques. (Of course this SAXS experiment, like the other techniques, does not examine the protein in the context of its normal biological molecular contacts in the cell.) The comparison of the SAXS results on squid HMM with and without Ca^2+^ (Fig. 2) confirm that the relatively compact conformation of the myosin off state is not an artifact of technique but is an intrinsic feature of this motor protein. Moreover, as was indicated by sedimentation studies (for scallop HMM) [16], the SAXS studies of squid HMM also show that the Ca^2+^–free off-state conformation is attained whether in the presence of ADP or an ATP analogue (Fig. 2b). While Guinier analysis indicates that the samples are not perfectly monodisperse, comparison of the calculated scattering profile of the known electron microscopy structure of tarantula HMM with the measured “off” state profile of squid HMM shows unambiguous agreement. Pair distance distribution functions derived from the inverse Fourier transform method show a dramatic change in shape upon binding Ca^2+^ that is reflected in the change in R_g_ and D_max_ parameters as well. Based on molecular weight estimates from two independent methods, it is unlikely that the structural differences seen here can be attributed to aggregation. Squid HMM folds into a compact state very similar to tarantula HMM in the absence of Ca^2+^.

### Evolutionary implications

In addition to confirming the relatively compact nature of the off state in solution, these results also bear on the evolutionary history of myosin-linked regulation. One general question in the field of evolutionary biology is whether a particular trait observed in more than one species was acquired independently in those species, through convergent evolution, or inherited from the common ancestor, during the divergent evolution of the populations. The latter is often assumed, e.g. as in reference [12], but still needs to be supported by analysis. The presence of striated muscle in bilaterians (e.g., humans) and cnidarians (e.g., jellyfish), for example, now appears to be a result of independent evolution, according to an analysis of the molecular components of this muscle in disparate species [24].

Another method by which one type of evolution may be distinguished from the other is based on the frequency with which a trait is found in descendants of an ancestor. A trait found in the vast majority or all descendants of a common ancestor likely also occurred in the common ancestor, rather than having been acquired separately and recently in each species. Such a conclusion is derived from the principle of parsimony [25], where minimizing the total amount of evolutionary change would argue in this case for acquisition of the trait once in the ancestor species followed by inherited divergent evolution. On the other hand, a trait found in a minority of distantly related descendants, without being found in other close relatives, likely did evolve separately in each of them [25].

**Figure 4:**
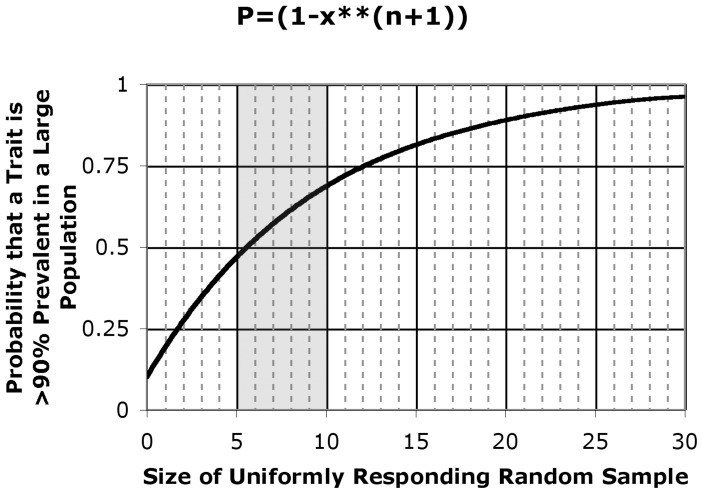
Probability that a feature is conserved as a function of sample size. The observation that squid HMM is relatively compact in the off state, together with this same observation in each of the other myosins previously examined (see text), increases the probability that this conformation is conserved in myosin-linked regulation. The formula for probability (P), shown on the y-axis, as a function of the size of a sample for which all members share the conformational feature, was developed by Ji Li (see Methods). Shaded region reflects the approximate number of independent species for which the compact conformation of off-state myosin has been observed (see text).

To determine whether a trait is nearly universal or not, without actually examining each descendent species, it is possible to perform a “poll” on a random sample of diverse species. In this case, sample size is critical. The observation that each member of a random sample exhibits a particular trait does not allow one to predict with high confidence that the trait is prevalent in all (or most of) the descendants if the sample size is small, say consisting of fewer than about 6 cases (Fig. 4). By contrast, as the sample size increases, and the trait continues to be observed in each of the samples, confidence increases that the trait is truly prevalent among the descendent species.

Such is the result of our SAXS studies on the regulatory structure of squid myosin. Off-state regulated myosin (HMM, free myosin, and/or thick filament) from two closely related vertebrate sources (chicken and turkey smooth muscle [9], [10], [11], [12], [17]), three arthropod sources (tarantula[2], [13], scorpion (see Ref. [15]), and limulus [2], [14]) — and now two mollusks (scallop [12], and squid), have all shown features of a relatively compact structure. (Unregulated myosins from mouse skeletal and cardiac muscle can also form the compact structure, albeit in a more labile form [2].) The current data from squid HMM provide additional evidence (Fig. 4) that a folded/compact structure is a characteristic feature of the inhibited state of myosins, and that this feature has been passed down through hundreds of million of years of divergent evolution.

## Materials and Methods

### Protein isolation

Squid (*Loligo peali*) were obtained from the Marine Biological Laboratory, Woods Hole, MA., where the funnel retractor muscle was harvested. Myosin was prepared as described [26]. Squid myosin was digested to HMM as described by Stafford et.al. (2001) with modifications [23]. All protein work was performed at 4°C unless otherwise specified. Protein purity was analyzed at all steps by SDS-PAGE gels [27] stained with Coomassie Brilliant Blue R250. The total molecular weight of this squid HMM as observed on these gels is ∼360 kDa.

### Protein purification

Hydroxylapatite (HA) column purification of the crude HMM was performed as described [23] using Calbiochem Hydroxylapatite, Fast Flow. The HMM portion eluting at 100 mM potassium phosphate (K.Pi) (pH 7.2–7.4) looks pure on gels and gives high yield, but also contains some high molecular weight contamination; this portion does not crystallize well (even after sizing column purification), but still has greater than 90% sensitivity and normal to high ATPase activity (0.3–0.5 moles/(min*mg)) measured with the coupled assay method [28]. This material was flash-frozen and stored long term in liquid nitrogen, and later purified further by a sizing column run for the SAXS experiments.

### Final Sample Preparation

Previously frozen HA-purified HMM (100 mM K.Pi) was thawed for five minutes in a room temperature water bath, then run over a Pharmacia Superdex 200 column at room temperature in: [40 mM Na Malonate (pH 7), 20 mM 4-(N-Morpholino)butanesulfonic acid (MOBS) (pH 7), 3 mM NaN_3_, 2.5 mM MgCl_2_, 0.5 mM Tris(2-carboxyethyl)-Phosphine Hydrochloride (TCEP), 0.5 µg/ml leupeptin]. Pure fractions were combined, and 0.2 mM AMP.PNP was added.

This sample was divided in half, and 1 mM EGTA was added to the “off” sample. This was concentrated (Centricon 10), AMP.PNP was increased to 2 mM, and the sample was clarified (80,000 g for 30 minutes). Protein concentration = 2.25 mg/ml, determined by the Bradford method (using BioRad Protein assay solution). The final AMP.PNP and EGTA molarities were added to a small portion of the column buffer for SAXS dilutions.

The other half of the 0.2 mM AMP.PNP pure fraction combination described above was used for the on state sample. After addition of 0.5 mM CaCl_2_, this sample was concentrated (Centricon 10). AMP.PNP was increased to 2 mM final, and the sample was clarified (80,000 g for 30 minutes). Protein concentration = 2.2 mg/ml. The final AMP.PNP and CaCl_2_ molarities were added to a small portion of the column buffer for SAXS dilutions.

Glucose isomerase used as the SAXS molecular weight standard (Hampton Research, Aliso Viejo, CA) was prepared in 10 mM HEPES buffer at pH 4.0 with 1 mM MgCl_2_ at 1.25 mg/ml. The concentration of the glucose isomerase standard was determined by the A280 method using the reported extinction coefficient of 45660 M^−1^cm^−1^ [29]. The Bradford method is generally regarded as more accurate than the A280 method; consequently, molecular mass estimates based on this glucose isomerase standard will be dominated by expected error levels for the A280 method (5–10%). Error levels of 10% have been reported in the literature for SAXS mass estimates using A280 on a wide range of proteins [30]. Error estimates reported in the Results section, however, are standard deviations of multiple measurements reported in Table S1.

### SAXS data collection

All samples were centrifuged at 14,000 RPM for ten minutes prior to data collection. Each sample was prepared at three concentrations (3/3, 2/3, and 1/3 dilution of maximum) to assess concentration dependence. SAXS data were collected on CHESS beamline F2 at 9.881 keV (1.2563 Å, the tantalum edge) at 1×10^10^ photons/s. The X-ray beam was collimated to 250×250 µm^2^ diameter and centered on a 2 mm diameter vertical quartz capillary tube with 10 µm thick walls (Hampton Research, Aliso Viejo, CA). The capillary tube and full X-ray flight path, including beamstop, were kept *in vacuo* to eliminate air scatter. Sample plugs of approximately 15–20 µl were delivered from a 96-well plate to the capillary using a Hudson SOLO single-channel pipetting robot (Hudson Robotics Inc. Springfield, New Jersey). To reduce radiation damage, sample plugs were oscillated in the X-ray beam using a computer-controlled syringe pump (Aurora Biomed, Vancouver, B.C., Canada). Images were collected on a Pilatus 100K-S detector (Dectris, Baden, Switzerland) with sequential 180 s exposures being used to assess possible radiation damage. Sample and buffer solutions were normalized to equivalent exposure before subtraction using beamstop photodiode counts. The complete automated BioSAXS system is described elsewhere[31].

Sample-to-detector distance was calibrated using silver behenate powder (The Gem Dugout, State College, PA). Images were reduced to profiles and buffer subtracted using the BioXTAS RAW software [18]. The useful q-space range (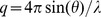 with 2θ being the scattering angle) was generally from q_min_ = 0.01 Å^−1^ to q_max_ = 0.27 Å^−1^.

Radius of gyration (R_g_) was calculated using both the method of Guinier [32] and the inverse Fourier transform (IFT) method as implemented in the GNOM program [20]. In the case of the Guinier method, linear fitting was performed on data having a range of 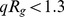 unless otherwise noted. Scattering profiles are available as Supplemental Data HMM_CA S1 and HMM_EGTA S2. The profiles are supplied as 3-column plain text files (q, I, err) readable by EMBL ATSAS and BioXTAS RAW software. Interactive fitting was performed using the BioXTAS RAW program [18]. Molecular weights were estimated by two independent methods. First, the intensity of a standard (glucose isomerase) at zero scattering angle I(0) was determined by Guinier analysis and used to estimate molecular weight by comparison [30]. Second, a concentration-independent method utilizing the Porod invariant was applied through the SAXS MoW server (www.if.sc.usp.br/~saxs/) [19].

The Inverse Fourier Transform (IFT) method implemented by GNOM was run multiple times over a range of possible values for the maximum diameter (D_max_) with total estimate scores[33] and χ^2^ values being examined at each step. The maximum diameter of the protein is not a well-defined quantity, especially in the presence of mixtures of conformations, higher-order oligomers, or aggregates. Values of D_max_ chosen for comparison of P(r) functions were the smallest values that yielded smooth decay of the curve to zero while staying below the Shannon limit set by the experimental apparatus: D_max_<π/q_min_.

The widely used CRYSOL program was employed to evaluate how well atomic models agree with observed scattering profiles [34]. Shape reconstruction was performed by running 10 independent DAMMIF calculations [35] and building a consensus envelope using the DAMAVER program [36]. Alignment of envelopes with the coordinate model was performed using the SUPCOMB program [37]. (See Methods S1.)

### Derivation of Conditional Probability

How likely is it that the compact conformation of off-state myosin is highly conserved? Figure 4 provides an answer to this question by plotting the conditional probability 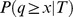, which is the probability P that the proportion q of all regulated myosins that form a compact conformation in the off state is greater than or equal to x (e.g., 0.90), given the conditional event T that a compact conformation is observed in all of n randomly sampled cases. In the figure, the probability is plotted on the y-axis and n is plotted on the x-axis. To solve for this conditional probability, first note that the probability of each individual regulated myosin adopting a compact off state is q, and the event T thus occurs with the probability 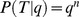. Therefore,
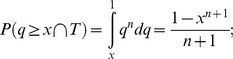
Similarly,
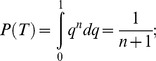
Finally,
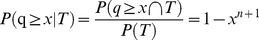



## Supporting Information

Data S1 Small angle x-ray solution scattering profile for squid heavy meromyosin+Ca2++AMP.PNP. The profile is plain text in three-column format (q, I, err).
(DAT)

Data S2 Small angle x-ray solution scattering profile for squid heavy meromyosin+EGTA+AMP.PNP. The profile is plain text in three-column format (q, I, err).
(DAT)

Figure S1 Guinier plots of HMM samples in the presence and absence of Ca2+. Both samples show some systematic deviation from linearity at lowest q, which indicates the presence of larger components (aggregation or higher-order oligomers) in the preparation. All low q values are shown in the Guinier plots. In all cases except the most dilute profile, qRg≈1.3 for the highest q value used in the analysis. The three lowest q values in the HMM+EGTA+AMP.PNP profile were excluded from the fitting to minimize the influence of the apparent aggregation.
(TIFF)

Figure S2 Superposition of the Guinier curves for the highest concentrations of both “on” and “off” states. Significant linear regions exist in both profiles despite some apparent aggregation and the Rg values are significantly different. Note that the HMM+EGTA+AMP.PNP profile has been shifted down by a small arbitrary increment for clarity of comparison.
(TIFF)

Figure S3 Kratky plots for Squid HMM in both the absence (HMM+EGTA+AMP.PNP) and presence (HMM+Ca2+AMP.PNP) of Ca2+. The fall-off of the tails in the Porod region (high q) indicates that both states are folded.
(TIFF)

Figure S4 Comparison of experimental data to inverse Fourier solutions for the HMM+EGTA+AMP.PNP. Two different choices for the maximum diameter are shown here (Dmax = 200 Å, 295 Å), but the {back transformed) inverse Fourier solutions are nearly the same within the range sampled by the experimental data. The slight deviation from linearity seen in the small-angle portion of the Guinier plot (Fig. S2) appears here as the slight positive deviation of the data (black) from the back-transformed P(r) curves (green and dashed red).
(TIFF)

Figure S5 Comparison of experimental data to inverse Fourier solution for HMM+Ca2+AMP.PNP.
(TIFF)

Figure S6 Alignment of homologous tarantula HMM structure with envelope for HMM+EGTA+AMP.PNP (blue). The HMM+Ca2++AMP.PNP state is similar, but somewhat more extended (pink).
(TIFF)

Figure S7 Experimental data for HMM+EGTA+AMP.PNP (blue) superimposed on the dummy atom (DAMMIF) model (red).
(TIFF)

Figure S8 Experimental data for HMM+Ca2+AMP.PNP (blue) superimposed on the dummy atom (DAMMIF) model (red).
(TIFF)

Methods S1 Detailed procedure, parameters and references for inverse Fourier transform calculations and molecular shape reconstructions of squid heavy meromyosin samples.
(DOCX)

Table S1 Guinier analysis parameters for all dilutions of HMM samples in the absence (HMM+EGTA+AMP.PNP) and presence (HMM+Ca2+AMP.PNP) of Ca2+ ions. Data are given for the first and second successive 180 s x-ray exposures (exposure 1 and exposure 2 respectively) when available. Parameter qStart gives the smallest q value in the fitting range. The product qRg is the value calculated at the maximum q value used in the fitting range. The parameter rsq is goodness of fit and I(0) is the intensity extrapolated to q = 0. Rg values computed for the inverse Fourier Transform (IFT) method are the real-space values reported by the program GNOM 1.
(DOCX)
