# Supplementary figures and images for: X-Ray Solution Scattering of Squid Heavy Meromyosin: Strengthening the Evidence for an Ancient Compact off State
Source: PLoS One. 2013 Dec 17;8(12):e81994. doi: 10.1371/journal.pone.0081994 (PMC3866118; doi:10.1371/journal.pone.0081994)

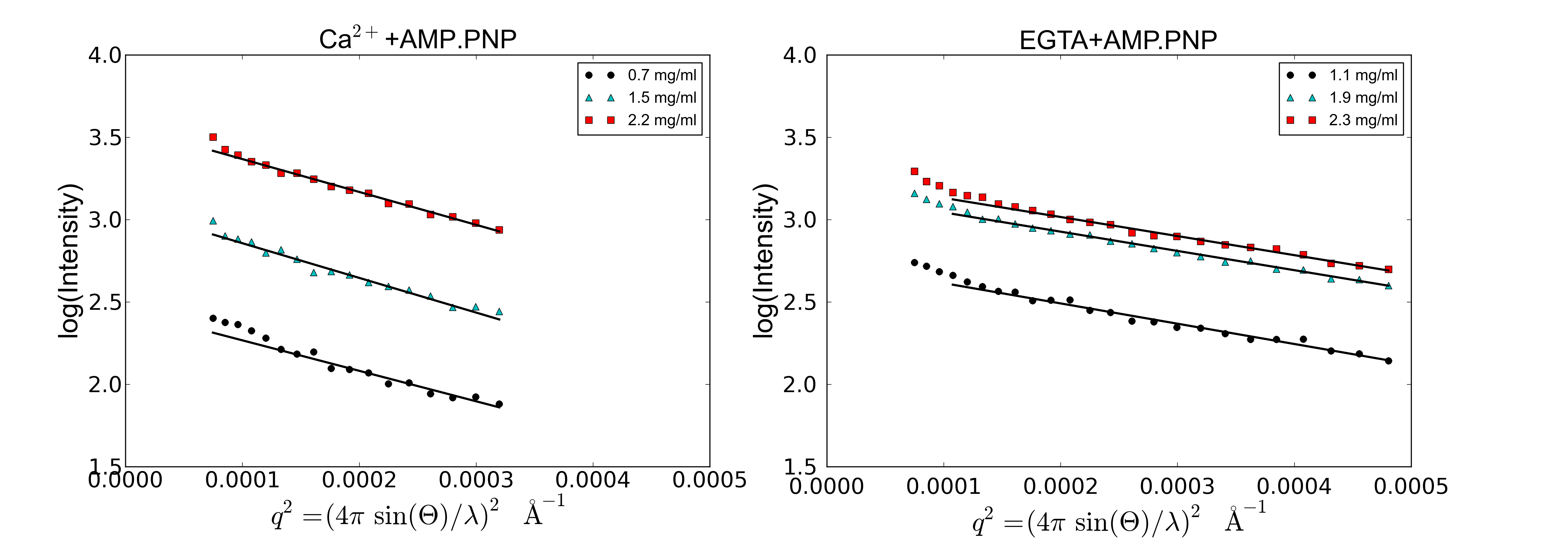

Supplement: Figure S1 — Guinier plots of HMM samples in the presence and absence of Ca2+. Both samples show some systematic deviation from linearity at lowest q, which indicates the presence of larger components (aggregation or higher-order oligomers) in the preparation. All low q values are shown in the Guinier plots. In all cases except the most dilute profile, qRg≈1.3 for the highest q value used in the analysis. The three lowest q values in the HMM+EGTA+AMP.PNP profile were excluded from the fitting to minimize the influence of the apparent aggregation. (TIFF) [file pone.0081994.s003.tiff]

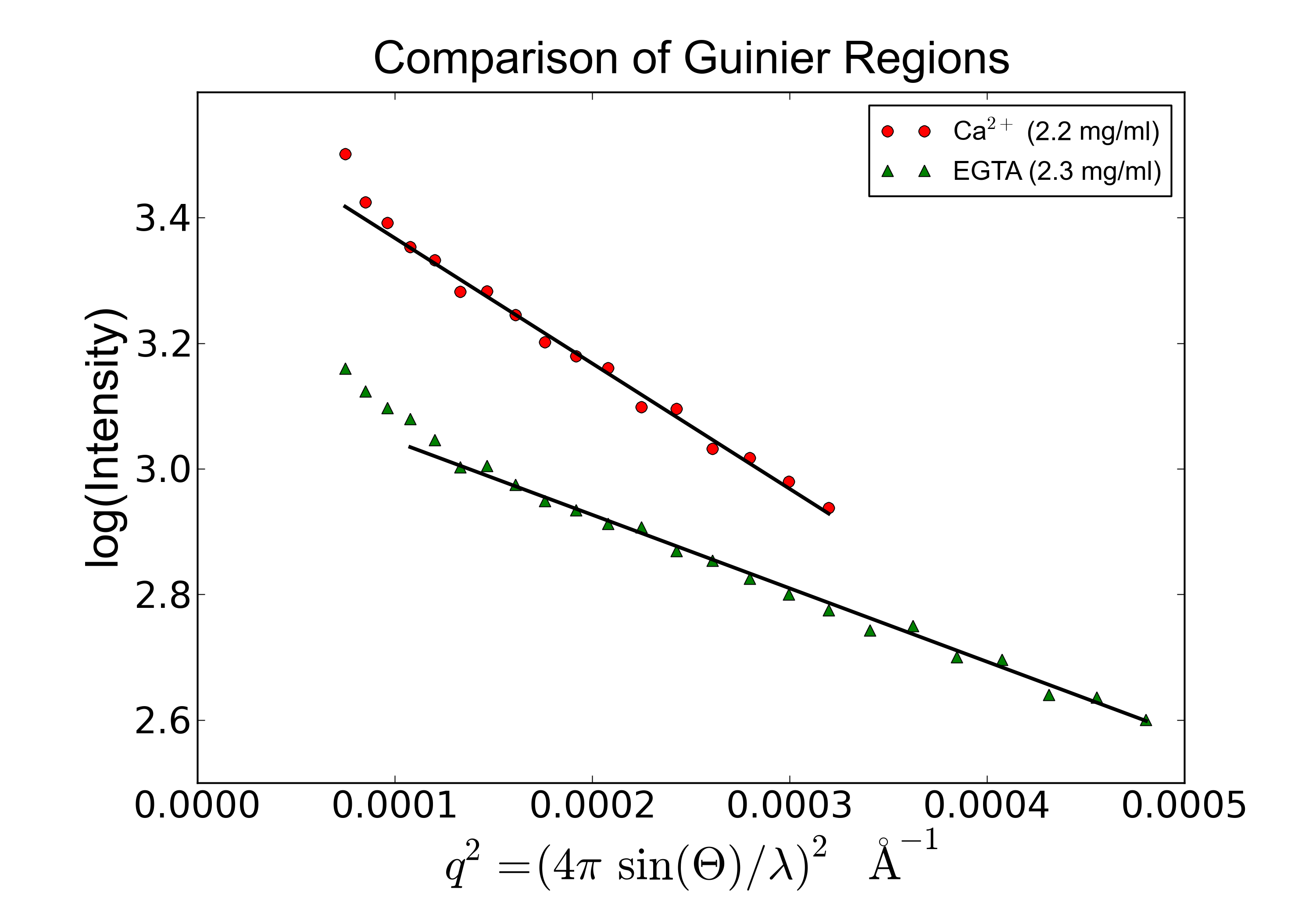

Supplement: Figure S2 — Superposition of the Guinier curves for the highest concentrations of both “on” and “off” states. Significant linear regions exist in both profiles despite some apparent aggregation and the Rg values are significantly different. Note that the HMM+EGTA+AMP.PNP profile has been shifted down by a small arbitrary increment for clarity of comparison. (TIFF) [file pone.0081994.s004.tiff]

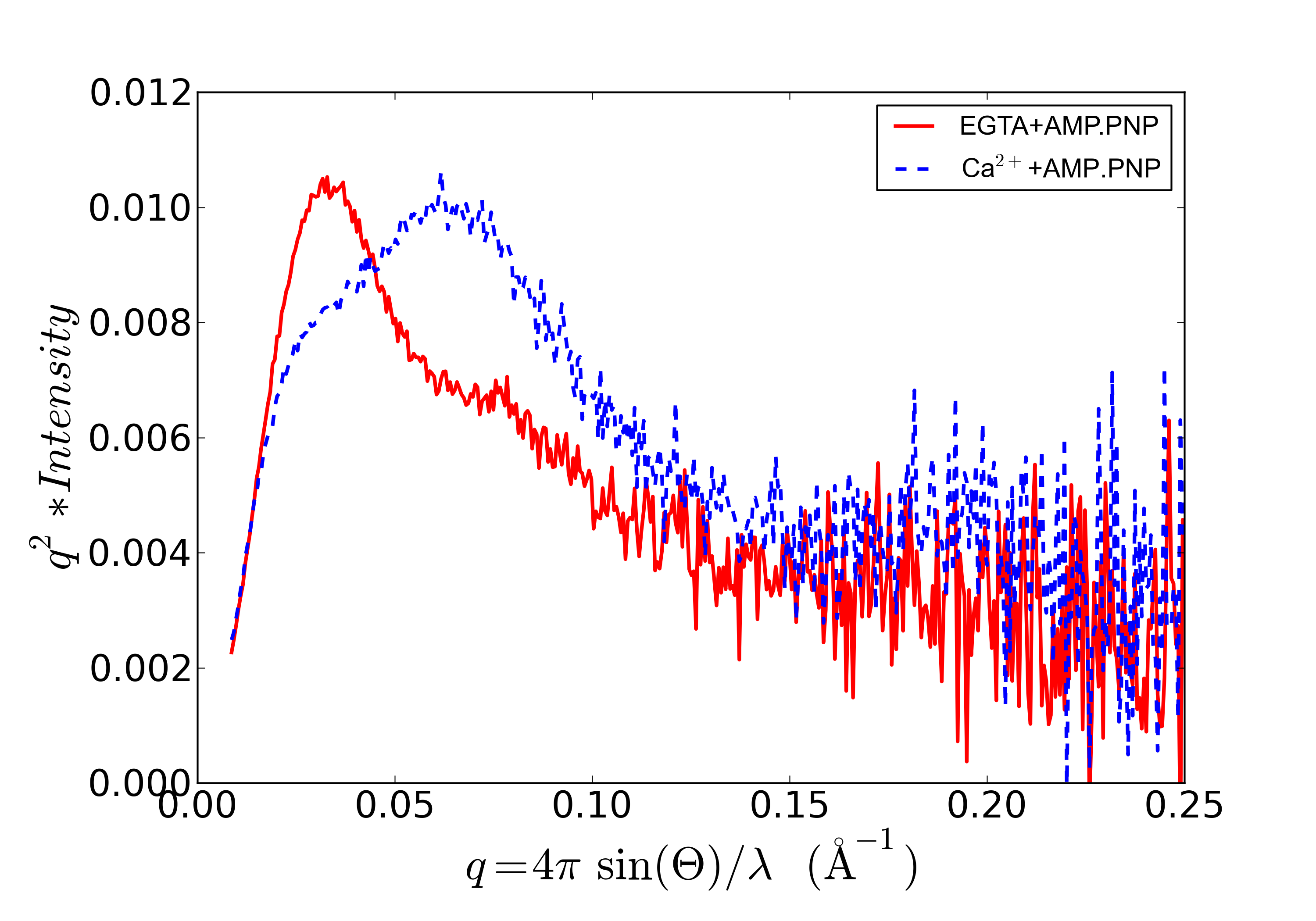

Supplement: Figure S3 — Kratky plots for Squid HMM in both the absence (HMM+EGTA+AMP.PNP) and presence (HMM+Ca2+AMP.PNP) of Ca2+. The fall-off of the tails in the Porod region (high q) indicates that both states are folded. (TIFF) [file pone.0081994.s005.tiff]

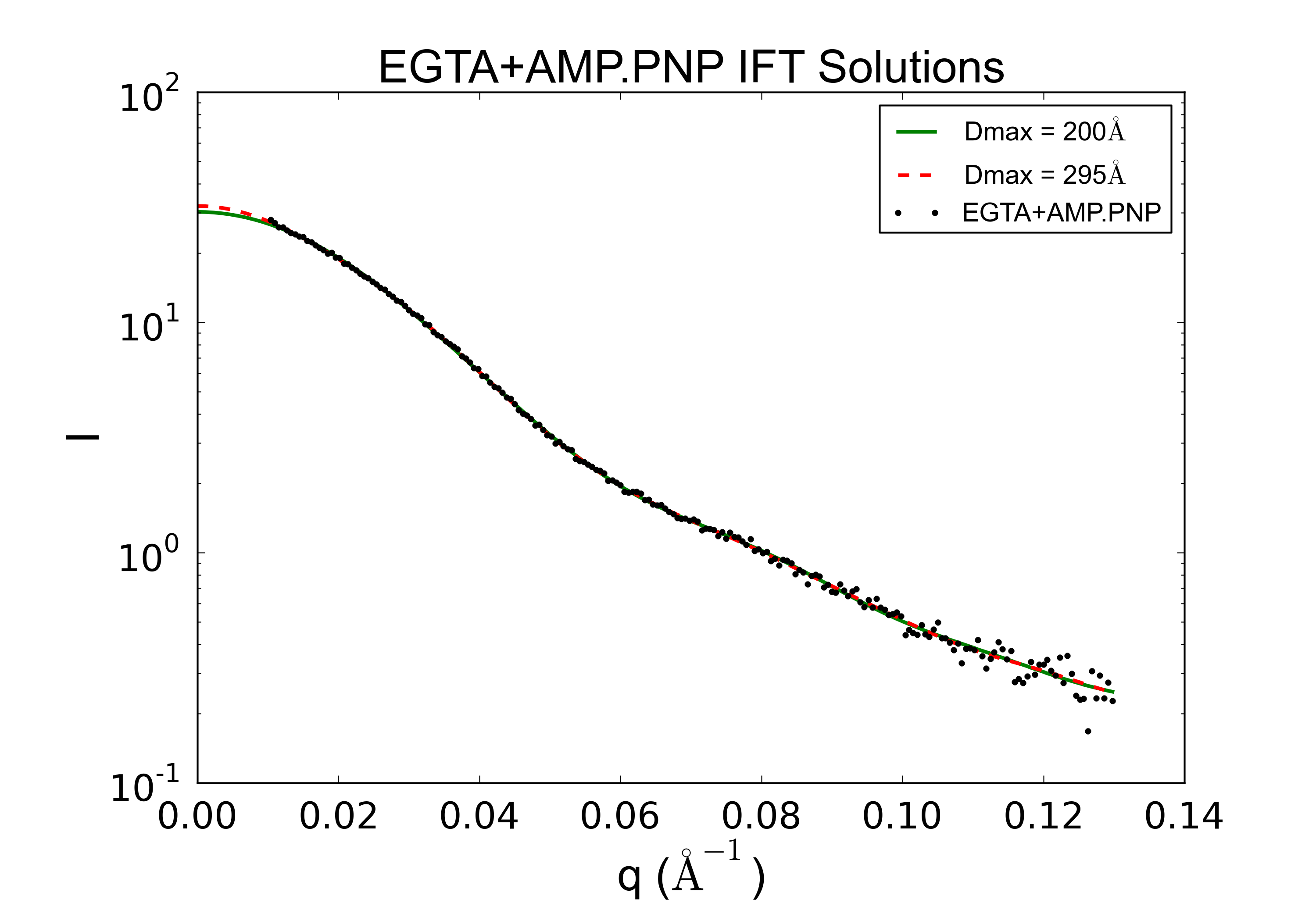

Supplement: Figure S4 — Comparison of experimental data to inverse Fourier solutions for the HMM+EGTA+AMP.PNP. Two different choices for the maximum diameter are shown here (Dmax = 200 Å, 295 Å), but the {back transformed) inverse Fourier solutions are nearly the same within the range sampled by the experimental data. The slight deviation from linearity seen in the small-angle portion of the Guinier plot (Fig. S2) appears here as the slight positive deviation of the data (black) from the back-transformed P(r) curves (green and dashed red). (TIFF) [file pone.0081994.s006.tiff]

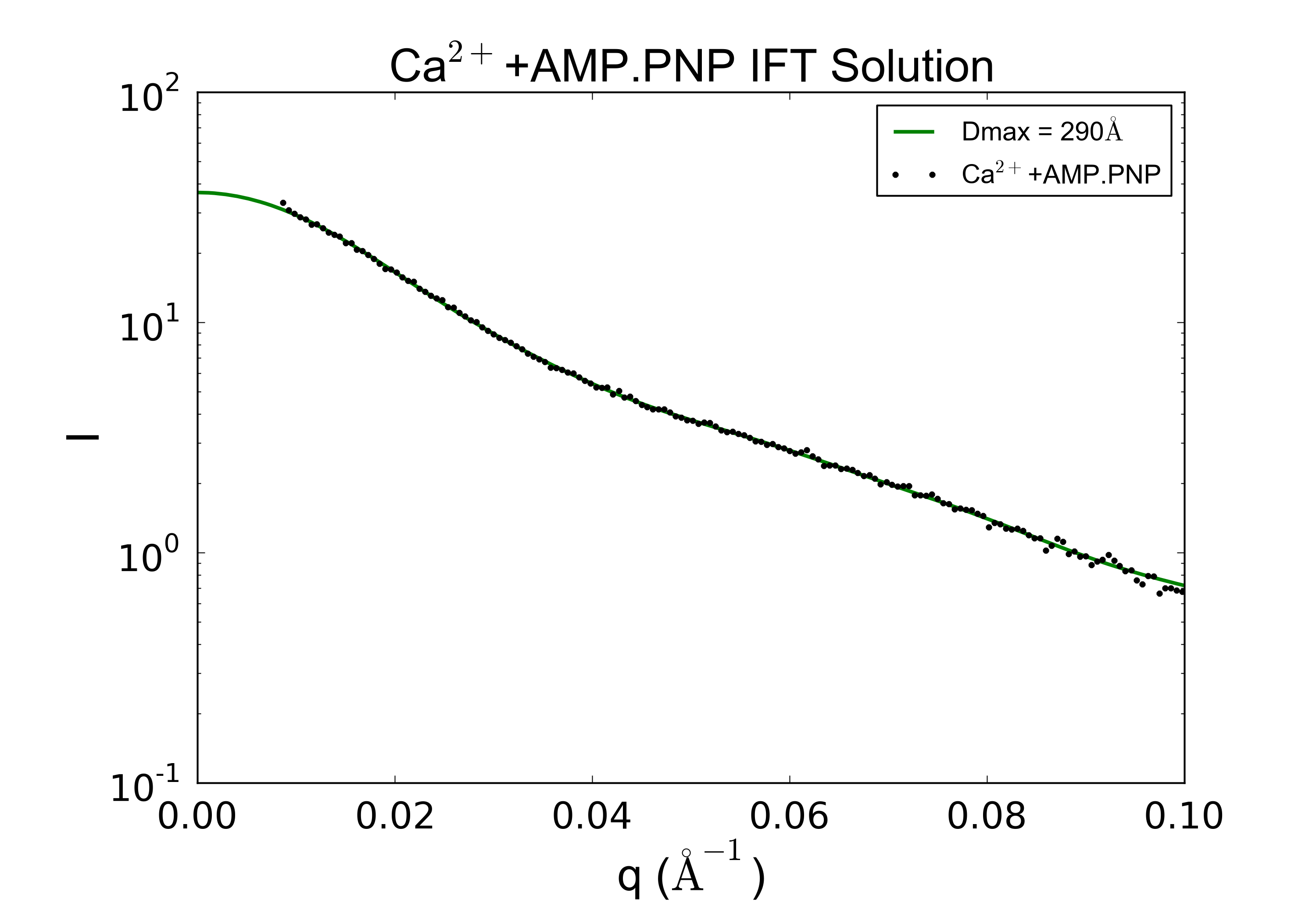

Supplement: Figure S5 — Comparison of experimental data to inverse Fourier solution for HMM+Ca2+AMP.PNP. (TIFF) [file pone.0081994.s007.tiff]

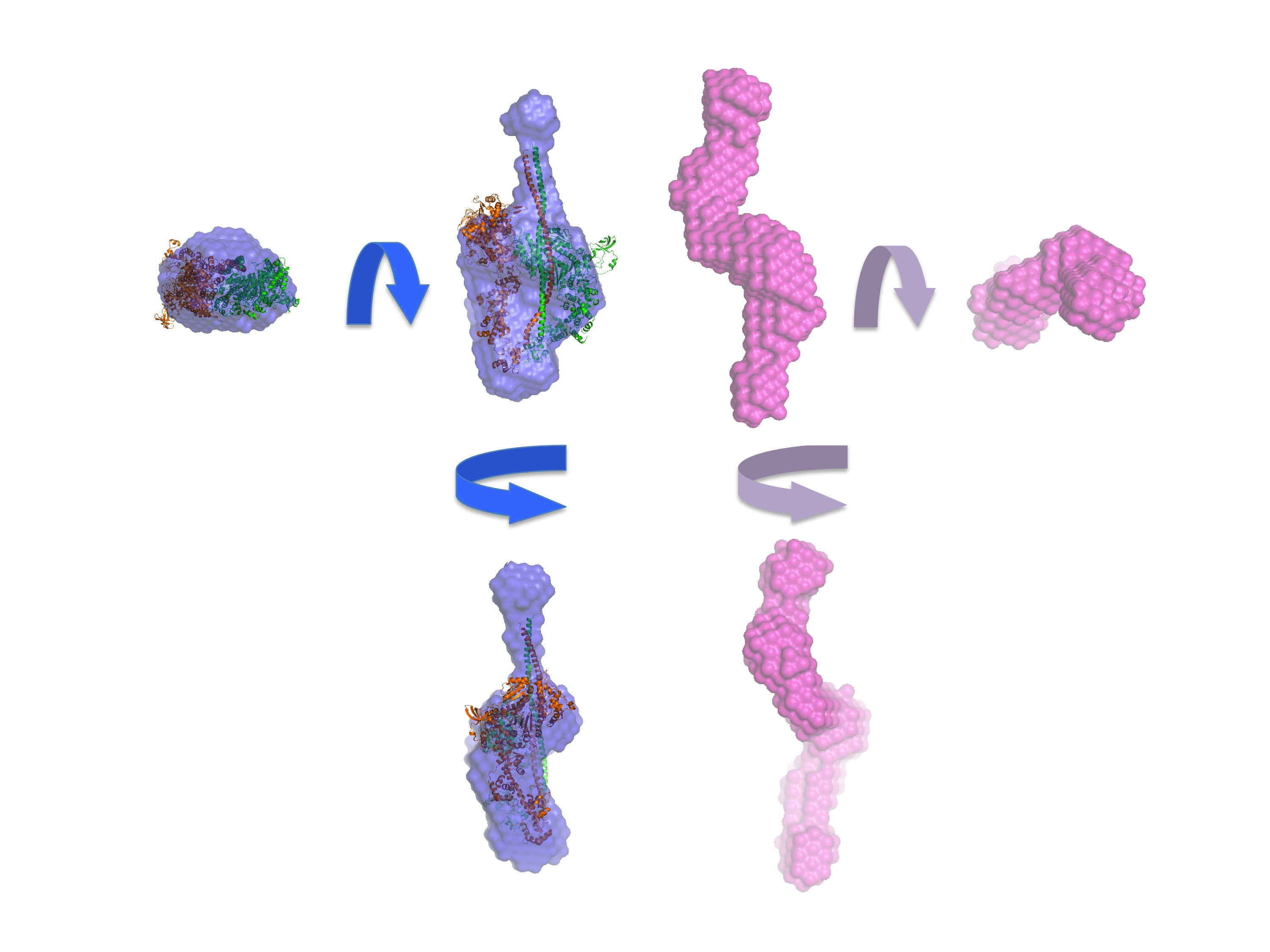

Supplement: Figure S6 — Alignment of homologous tarantula HMM structure with envelope for HMM+EGTA+AMP.PNP (blue). The HMM+Ca2++AMP.PNP state is similar, but somewhat more extended (pink). (TIFF) [file pone.0081994.s008.tiff]

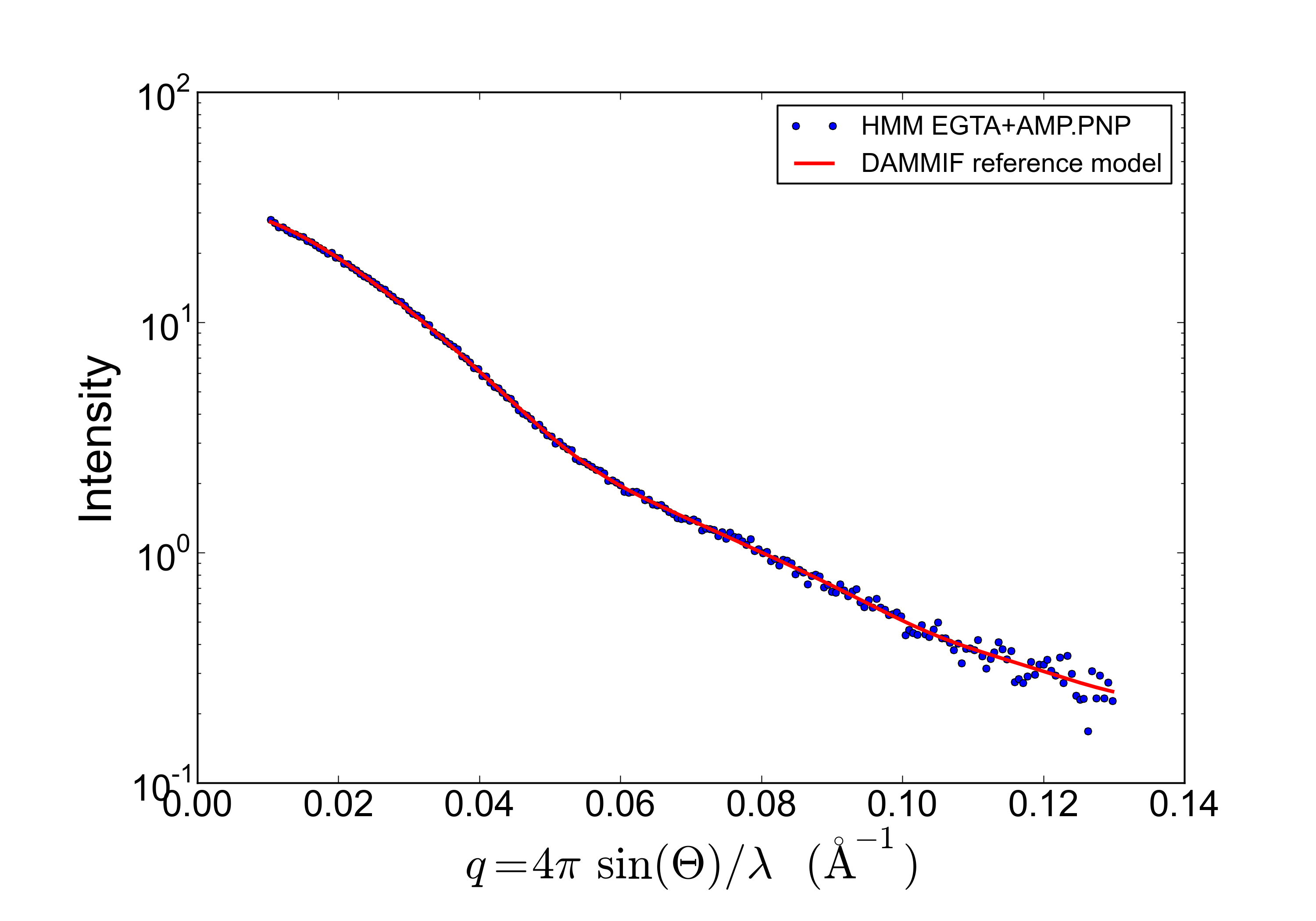

Supplement: Figure S7 — Experimental data for HMM+EGTA+AMP.PNP (blue) superimposed on the dummy atom (DAMMIF) model (red). (TIFF) [file pone.0081994.s009.tiff]

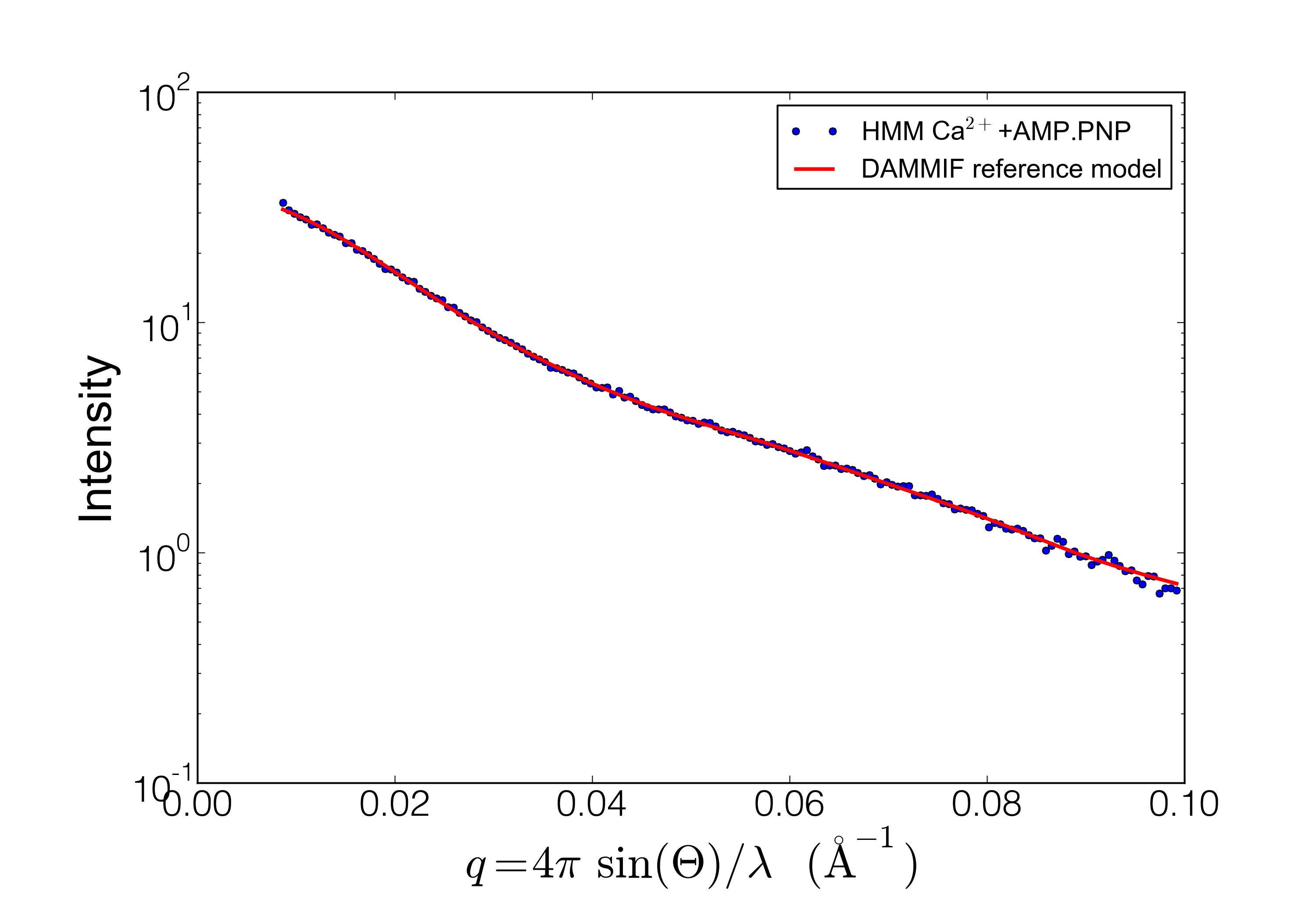

Supplement: Figure S8 — Experimental data for HMM+Ca2+AMP.PNP (blue) superimposed on the dummy atom (DAMMIF) model (red). (TIFF) [file pone.0081994.s010.tiff]
