# Supplementary material for: X-Ray Solution Scattering of Squid Heavy Meromyosin: Strengthening the Evidence for an Ancient Compact off State
Source: PLoS One. 2013 Dec 17;8(12):e81994. doi: 10.1371/journal.pone.0081994 (PMC3866118; doi:10.1371/journal.pone.0081994)
Supplement: Methods S1 — Detailed procedure, parameters and references for inverse Fourier transform calculations and molecular shape reconstructions of squid heavy meromyosin samples. (DOCX) [file pone.0081994.s011.docx]

**X-ray Solution Scattering of Squid Heavy Meromyosin: Strengthening the Evidence for an Ancient Compact Off State**

**Supplemental Methods**

**Richard E. Gillilan*^1^, V. S. Senthil Kumar*^2^, Elizabeth O’Neall-Hennessey^2^, Carolyn Cohen^2^, and Jerry H. Brown^2^**

Corresponding author: Jerry H. Brown: email: jhbrown@brandeis.edu

Phone: 1-781-736-2495; Fax: 1-781-736-2419

*These authors contributed equally to the work in this manuscript

^1^ Macromolecular Diffraction Facility, Cornell High Energy Synchrotron Source, Ithaca, New York, USA.

**^2^**Rosenstiel Basic Medical Sciences Research Center, Brandeis University, Waltham, Massachusetts, USA

**Inverse Fourier transform details**

The maximum diameter object (Dmax) observable in this experiment is set by the Shannon Limit π/qmin = 312 Å which is determined by the minimum observable q value. The HMM+EGTA +AMP.PNP calculation based on a Dmax value of 200 Å yields an “excellent” solution as reported by GNOM with a total estimate score of 0.904. The low-resolution shape, however, matches the proposed structure better when Dmax = 295Å. At this larger diameter, GNOM ranks the total estimate score of 0.705 as still “reasonable.” Quality of both solutions are indicated by the quality of the fit of the measured data to the profile obtained from the smooth P(r) function back-transformed into reciprocal space as shown previously in Figs. S4 and S5.

The HMM Ca2^+^ calculation was based on a Dmax value of 290 Å which yields a “good” solution with of total estimate 0.794. In either case, the “VALCEN” component of the total estimate for larger Dmax values was most responsible for the lower overall scale.[^1^](#_ENREF_1) This “perceptual” component naturally down-weights bimodal P(r) distributions as well as distributions with longer tails.

For the purpose of shape reconstruction, we have followed the widely used convention of truncating data at q_max_ = 8/R_g_. This truncation is done for the benefit of the DAMMIF algorithm which functions best with limited wide-angle data due to algorithmic approximations[^2^](#_ENREF_2).

**Shape reconstructions**

Shape reconstructions using the program DAMMIN were repeated 10 times, each with different starting conditions. The DAMAVER script was used to build a consensus shape and to generate statistics (Fig. S6). The off state, HMM+EGTA+AMP.PNP, with Dmax = 200 Å gives a mean normalized spatial discrepancy (NSD) value = 0.51 with one structure rejected. Choosing Dmax = 295 Å, the mean NSD value = 0.624 with no rejections. Both these values are well within the 0.5-0.7 range generally considered acceptable[^3^](#_ENREF_3). The Dmax = 200 Å choice gives a SQRT(Chi)= 1.218 agreement between experimental data and the reference dummy atom model. Dmax = 295 Å gives a slightly better SQRT(Chi) = 1.130. The model curves for both distances are indistinguishable for all but the first few points, but the Dmax = 295 Å gives a better fit to the published structure of tarantula HMM.[^4^](#_ENREF_4) The slightly larger Dmax apparently provides room for the extended helices in the structure. The alignment of the structure with the envelope was performed with the SUPCOMB program.[^5^](#_ENREF_5)

HMM Ca^2+^ + AMP.PNP at Dmax = 290 gives a borderline mean NSD = 0.802 with one rejection. The reference structure in that case, though, gives an excellent fit with SQRT(Chi)= 1.008. Comparisons between experimental profiles and calculated (reference) bead model profiles show good agreement (Figs. S7 and S8).

**References**

1. Semenyuk, A. V. & Svergun, D. I. (1991). Gnom - a Program Package for Small-Angle Scattering Data-Processing. *Journal of Applied Crystallography* **24**, 537-540.

2. Franke, D. & Svergun, D. I. (2009). DAMMIF, a program for rapid ab-initio shape determination in small-angle scattering. *Journal of Applied Crystallography* **42**.

3. Volkov, V. V. & Svergun, D. I. (2003). Uniqueness of ab initio shape determination in small-angle scattering. *Journal of Applied Crystallography* **36**, 860-864.

4. Alamo, L., Wriggers, W., Pinto, A., Bartoli, F., Salazar, L., Zhao, F. Q., Craig, R. & Padron, R. (2008). Three-Dimensional Reconstruction of Tarantula Myosin Filaments Suggests How Phosphorylation May Regulate Myosin Activity. *Journal of Molecular Biology* **384**, 780-797.

5. Kozin, M. B. & Svergun, D. I. (2001). Automated matching of high- and low-resolution structural models. *Journal of Applied Crystallography* **34**, 33-41.
