# Supplementary material for: X-Ray Solution Scattering of Squid Heavy Meromyosin: Strengthening the Evidence for an Ancient Compact off State
Source: PLoS One. 2013 Dec 17;8(12):e81994. doi: 10.1371/journal.pone.0081994 (PMC3866118; doi:10.1371/journal.pone.0081994)
Supplement: Table S1 — Guinier analysis parameters for all dilutions of HMM samples in the absence (HMM+EGTA+AMP.PNP) and presence (HMM+Ca2+AMP.PNP) of Ca2+ ions. Data are given for the first and second successive 180 s x-ray exposures (exposure 1 and exposure 2 respectively) when available. Parameter qStart gives the smallest q value in the fitting range. The product qRg is the value calculated at the maximum q value used in the fitting range. The parameter rsq is goodness of fit and I(0) is the intensity extrapolated to q = 0. Rg values computed for the inverse Fourier Transform (IFT) method are the real-space values reported by the program GNOM 1. (DOCX) [file pone.0081994.s012.docx]

**Table S1** Guinier analysis parameters for all dilutions of HMM samples in the absence (HMM + EGTA + AMP.PNP) and presence (HMM + Ca2+ AMP.PNP) of Ca^2+^ ions. Data are given for the first and second successive 180 s x-ray exposures (exposure 1 and exposure 2 respectively) when available. Parameter qStart gives the smallest q value in the fitting range. The product qR_g_ is the value calculated at the maximum q value used in the fitting range. The parameter rsq is goodness of fit and I(0) is the intensity extrapolated to q=0. Rg values computed for the inverse Fourier Transform (IFT) method are the real-space values reported by the program GNOM [^1^](#_ENREF_1).

| Guinier Analysis Data | | | | | | IFT | |
| --- | --- | --- | --- | --- | --- | --- | --- |
| HMM + EGTA +AMP.PNP | qStart | qRg | rsq | Rg ( Å) | I(0) | Rg ( Å) | I(0) |
| 2.3 mg/ml exposure 1 | 0.012 | 1.33 | 0.99 | 59.2 | 30.24 | 59.7±0.2* | 30.18±0.08 |
| 2.3 mg/ml exposure 2 | 0.014 | 1.27 | 0.99 | 59.5 | 28.94 |  |  |
| 1.9 mg/ml exposure 1 | 0.013 | 1.29 | 0.99 | 58.9 | 25.73 |  |  |
| 1.1 mg/ml exposure 1 | 0.012 | 1.33 | 0.98 | 60.8 | 15.45 |  |  |
| 1.1 mg/ml exposure 2 | 0.012 | 1.31 | 0.99 | 61.2 | 14.96 |  |  |
| HMM + Ca2+ AMP.PNP |  |  |  |  |  |  |  |
| 2.2 mg/ml exposure 1 | 0.010 | 1.33 | 0.99 | 77.4 | 35.41 | 83.9 ±0.8 † | 36.64 ±0.3 |
| 2.2 mg/ml exposure 2 | 0.012 | 1.30 | 0.99 | 75.2 | 33.18 |  |  |
| 1.5 mg/ml exposure 1 | 0.010 | 1.33 | 0.97 | 79.5 | 21.48 |  |  |
| 0.7 mg/ml exposure 1 | 0.012 | 1.33 | 0.96 | 74.5 | 11.61 |  |  |
| 0.7 mg/ml exposure 2 | 0.010 | 1.40 | 0.95 | 78.7 | 11.88 |  |  |

* Dmax = 200 Å † Dmax = 290 Å
